# Impact at two years of an intervention on empowerment among medical care teams: study protocol of a randomised controlled trial in a large French university hospital

**DOI:** 10.1186/s12913-019-4724-7

**Published:** 2019-12-03

**Authors:** Baptiste Cougot, Jules Gauvin, Nicolas Gillet, Kalyane Bach-Ngohou, Johan Lesot, Isaac Getz, Xavier Deparis, Claire Longuenesse, Anne Armant, Emmanuelle Bataille, Brice Leclere, Ghozlane Fleury-Bahi, Leïla Moret, Dominique Tripodi

**Affiliations:** 1 Department of Occupational Medicine and Environmental Pathology, Nantes University Hospital, Nantes, France; 2 Department of Psychology, University of Tours, Tours, France; 3 EE1901 QualiPsy laboratory, University of Tours, Tours, France; 4 Department of Public Health, Nantes University Hospital, Nantes, France; 5 Department of Occupational Medicine, Nantes University Hospital, Nantes, France; 6 Department of Biochemistry, Nantes University Hospital, Nantes, France; 7 UMR 1235 INSERM TENS “The enteric nervous system in gut and brain disorders”, University of Nantes, Nantes, France; 8 SSTRN Service de Santé au Travail de la Région Nantaise, Nantes, France; 9 ESCP Europe Business School, Ecole Supérieure de Commerce de Paris Europe, Paris, France; 10 Army Center for Epidemiology and Public Health, University of Aix-Marseille, Marseille, France; 11 Department of Psychology, University of Nantes, Nantes, France; 12 EA4638 Psychology Laboratory of Pays de la Loire, University of Nantes, Nantes, France; 13 UMR 1246 INSERM SPHERE “MethodS in Patients-centered outcomes and HEalth ResEarch”, Universities of Nantes and Tours, Nantes, France

**Keywords:** Intervention, Empowerment, Leader empowering behaviours, Management, Motivation, Occupational health, Workplace quality-of-life

## Abstract

**Background:**

Empowerment of hospital workers is known as a key factor of organizational performance and occupational health. Nevertheless, empowering workers remains a real challenge. As in many traditional organizations, hospitals follow a bureaucratic model defined by a managerial culture of control and a stratified organization, which at once weaken professionals’ mastery of their work and hinder their commitment and performance. Based on the existing literature this protocol describes a new managerial and organizational transformation program as well as the study design of its effect on worker empowerment in a large French public hospital. The project is funded by the French Ministry of Health for a total of 498,180 €.

**Methods:**

This study is a randomized controlled trial conducted in a French university hospital complex (CHU). The CHU comprises 12 sub-centers (SC) with about 20 care units and 1000 employees each. Randomization is performed at SC level. The intervention lasts 12 months and combines accompaniment of healthcare teams, frontline managers and SC directors to empower first-line professionals in the experimental SC. Quantitative outcome measurements are collected over 2 years during mandatory check-ups in the occupational medicine department. The primary outcomes are structural and psychological empowerment, motivational processes, managerial practices, working conditions, health and performance. Mixed linear modeling is the primary data analysis strategy.

**Discussion:**

The protocol was approved by the CHU health ethics committee. The results of the analysis of the intervention effects will be reported in a series of scientific articles. The results will contribute to reflection on prevention and management policies, and to the development of Workplace Quality-of-Life. If the intervention is a success, the system will warrant replication in other SCs and in other health facilities.

**Trial registration:**

The study was retrospectively registered at ClinicalTrials.gov on July 4, 2019 (NCT04010773).

## Background

As in many other countries, the French health system has been under constant pressure to control its medical expenditure [1–3], which has favoured styles of hospital management that are mainly focused on economic aspects. However, over recent years, empirical evidence [4–7] has emerged, alongside questioning in the managerial spheres [8], on the interconnections between quality-of-life in the workplace, care quality and economic performances. In this perspective, the *Haute Autorité de la Santé* (HAS, French health authority) is today clearly encouraging the piloting of hospital performances by way of workplace quality-of-life (W-QoL) [9]. In this respect, the HAS [8] chooses to define W-QoL not as a collection of dimensions reflecting an optimal state of health, but rather as a participatory organisational approach: ‘This promotes the notion that the perceptions entertained by staff on W-QoL depend on their ability to put their job content into words and act on that content. It is in favour of a role of the staff and their representatives as players in the construction of solutions, in particular in the organisational field, alongside the management, the administration and experts’ (p.35).

This redefinition of W-QoL recalls research in the field of psychology [10–12] and management [13–16] on the theme of empowerment. Giving more latitude and support to collectives in the definition of their work increases the levels of identification and implication, which in turn favours performance and wellbeing. However the ethnographic literature shows that an evolution of this nature entails a radical cultural and structural shift, because it questions the distribution of power within a health facility and the representation that each protagonist (manager, administration, doctors, nurses etc.) has of his or her role [17–20].

This raises two questions: 1) how can organisations be accompanied in achieving this structural and cultural transformation? And 2) how can the impact of an accompaniment of this sort towards this new conception of W-QoL be measured? Whether in providing a theoretical basis for the intervention, or in operationalising the assessment and measurement of these processes, the models of structural empowerment and psychological empowerment have been taken up in north America as relevant paradigms.

Since the early days, in the 1970s and 1980s, empowerment has been seen as a multi-level concept [6, 10, 11]. On the individual level, Thomas & Velthouse (1990), [15, 16, 21] conceptualised psychological empowerment as the creation of a system of cognitions (beliefs and attitudes) based on experience (and its subjective interpretation), which can be likened to systems of expectations of an individual in relation to his or her abilities, level of implication and performances, and this in turn is predictive of attitudes and behaviours such as commitment to the job and actual performance. This involves the belief entertained by an individual that he/she 1) has an impact on the professional environment, 2) that he/she possesses the required professional skills, 3) that his/her professional actions are the result of his or her own will-power, and 4) that his/her work has meaning.

On the environmental level, structural empowerment [14, 22] for its part relates to the social, physical and organisational components of the environment that favour, predict or mediate the influence, control or power of social actors. Here the issues are access to 1) opportunities for professional development, 2) relevant information on the enterprise and its strategies, 3) adequate support to enable and contribute to personal development, and 4) the possibility of exerting an influence to obtain more resources (funds, equipment, human resources).

The effects of psychological [23] and structural empowerment in care facilities have been widely studied in the literature. Whatever the level of implementation, favourable effects are observed on job satisfaction [24–27], caregiver health [25, 28–31], the intention to stay in the facility [30, 32, 33], commitment [24, 34, 35], innovation [16, 21, 36], management efficiency [16], implication in unplanned pro-organisational behaviours [31, 37], care quality [4, 38, 39], and performance generally [4, 37]. The psychological and structural levels of empowerment are thus relevant intervention targets, liable to achieve an overall improvement in the performances of care facilities.

To our knowledge, there are six controlled interventional studies in care environments that have targeted empowerment among first-line professionals.

Four of these studies targeted interventions among individuals. In each case, volunteering subjects were included in groups distinct from their respective work teams, and they were asked to exchange their experiences and practices in the presence of a facilitator, for the purpose of fostering empowerment. Three of these interventions presented positive effects on psychological empowerment among the participants. They did not however demonstrate any effect on structural empowerment [40] nor on job satisfaction [40, 41]. These results show that it is worth collectively supporting elaborations by individuals on the determinants of their experiences and the identification of solutions, so as to foster psychological empowerment. But at the same times these studies point to the limitations of this type of intervention, which does not have any effect on access to empowerment structures via team processes, and management or organisation adjustments.

McPhee, Dahinten et al. (2013, 2014), [34, 42] developed a training and back-up protocol centred on front-line managers, aiming to promote pro-empowerment managerial practices. The study evidenced a positive effect on team implication at 1 year, partially mediated by structural empowerment. However, this intervention did not appear to improve psychological empowerment among caregivers, nor did it improve commitment among disengaged professionals. Yet the literature shows that psychological empowerment is the precursor of the internalisation of motivation and implication [12, 15]. In this perspective, in the absence of psychological empowerment, participants would not be able to internalise the values associated with the job and enhance their level of implication, while those who already identify with their jobs would increase their implication. Thus, although this study genuinely demonstrates the value of interventions at management level, it also shows the limitations of this mode of intervention for psychological empowerment, and *in fine* for the actual commitment of professionals.

In another study by Laschinger et al. (2012) [43], teams and their front-line supervisor were directly implicated in a process of collective problem-solving with the support of a facilitator. The managerial staff had the support of a chief nursing officer. The intervention improved structural empowerment at 6 months (psychological empowerment was not assessed). However, access to information and opportunities was not improved. Access to these empowerment structures clearly involves protagonists and areas of action outside the care team. For instance, access to opportunities requires human resources in the area of careers, mobility, training and administrative rules for promotion. Access to information also involves different facility management structures and their ability to communicate and transfer information to healthcare teams concerning the situation of the hospital, its values and its objectives. In this perspective, it can be postulated that focusing on healthcare teams and their local management, with the support of only a chief nursing officer, restricts the scope for action of a more structural nature by the management (general director, human resource director, Chief medical officer, and other types of management), despite the fact that these protagonists contribute to defining professionals’ access to empowerment structures.

In view of all these political, theoretical and empirical elements (e.g., ethnographical, experimental), the present study was designed to test the efficacy of a new intervention model combining an accompaniment of healthcare teams, training and accompaniment of front-line managers and top managers (directors), for the purpose of enhancing the powers of first-line professionals.

The design is an experimental randomised controlled study on the effect of an intervention in a university hospital Sub-Centre (the “intervention SC”) compared to a “control SC”, within a large French university hospital complex (*Centre Hospitalier Universitaire*, CHU) with more than 12,000 salaried staff.

The project is called CHRYSALIDE. It is funded on call for scientific tender by the French ministry of health for total of 498,180 €. The results of this research are intended to orient future public policies in the areas of prevention and health facility management.

This article describes the study protocol and includes a description of the intervention and the quantitative methods used to assess its effects. The study was planned to start in January 2017, recruitment of participants began in 2018 for the baseline, and the data collection is to be complete in summer 2020.

## Methods/design

### Study design

This study is a randomised controlled trial conducted in a large university hospital centre in France.

The psycho-social intervention lasts 12 months. It simultaneously targets healthcare teams, department management and SC management. The randomisation was therefore performed at SC level.

**Fig. 1:**
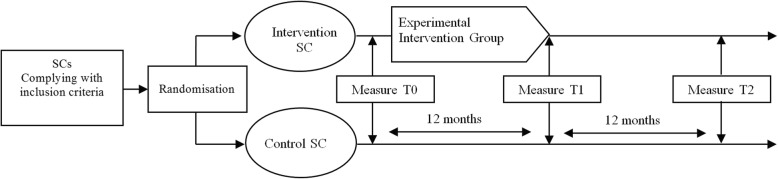
CHRYSALIDE Flowchart

The quantitative study of the effects of the intervention combines an assessment via self-administered questionnaire and the collection of clinical indicators in the course of a mandatory medical check-up in the occupational medicine department in the CHU. Three assessment times are planned: a baseline assessment, an assessment immediately after the intervention and an assessment 1 year after the end of the intervention (Fig. 1).

### Objectives

1. To assess the impact at one and 2 years of a standardised psychosocial intervention programme on the structural empowerment score in an “intervention SC”, in comparison with a “control SC”.
2. To assess the impact of the intervention at one and 2 years on psychological empowerment scores.
3. To detail the effects of the intervention on sub-dimensions of structural and psychological empowerment.
4. To assess the effect of the intervention on motivational processes and factors.
5. To assess the effect of the intervention on managerial practices.
6. To assess the impact of the intervention on working conditions, health and performance.

### Sub-centers recruitment

**Fig. 2:**
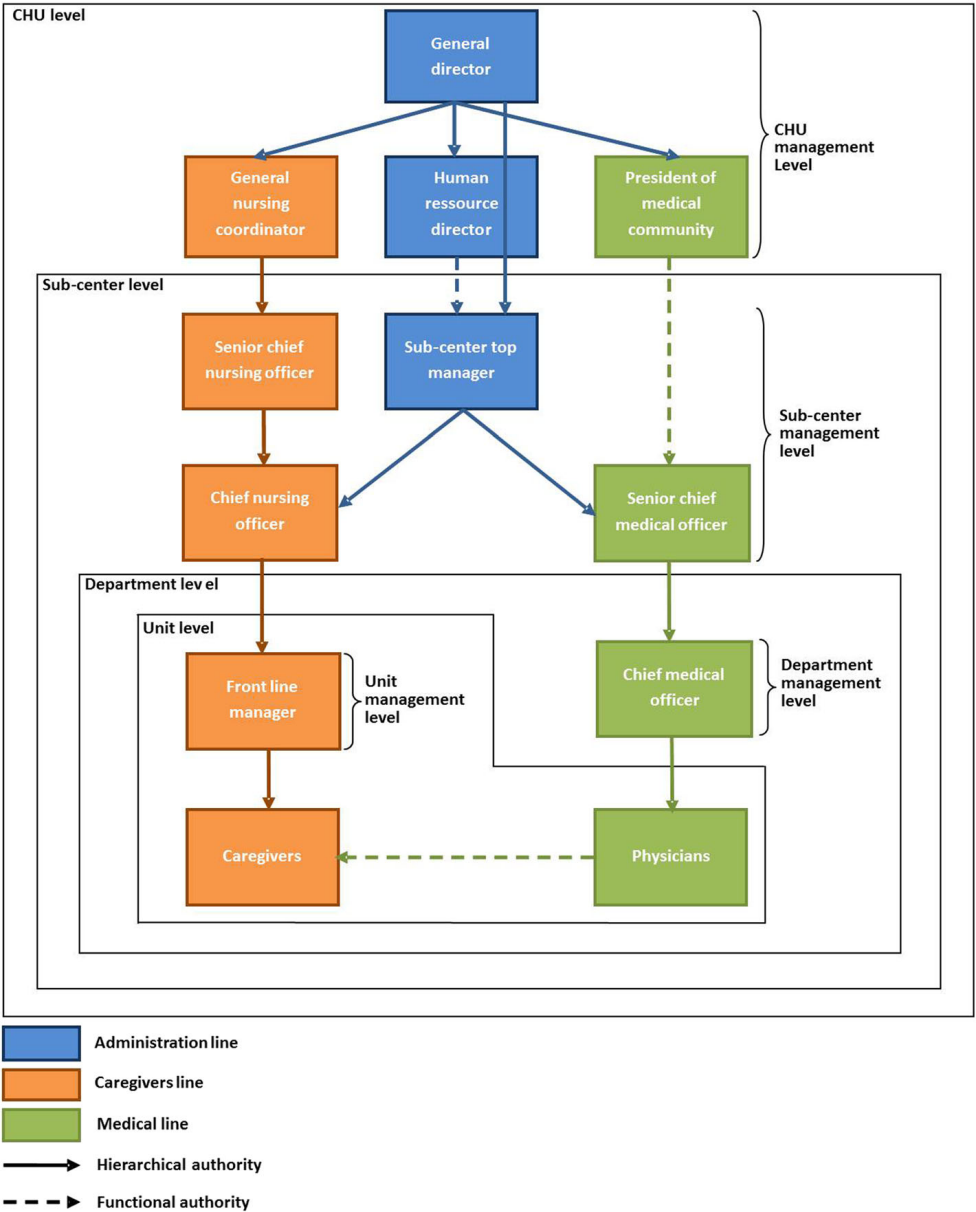
Hierarchical structure of the CHU

The targeted CHU comprises 12 SCs with five to six departments each, each in turn grouping a number of care units for a total of 20 to 25 per SC. The size of the SCs varies from 800 to more than 1000 salaried staff. The hierarchical structure is presented in Fig. 2.

The recruitment of SCs before randomisation was conducted in collaboration with CHU-level management according to the following eligibility criteria:

- At the CHU management level, agreement by the CHU General Director (*Directeur Général*, DG), the human resources director, the president of the medical community and the general nursing coordinator.
- No major reorganisation or re-affectation planned over the study period.
- At the SC management level, agreement by the SC top-manager, the senior chief nursing officer, the senior chief medical officer and the chief nursing officer.
- Both medical and surgical activity in the SC.

A first random selection was performed among the eligible SCs to determine the two SCs to be included in the study. The randomisation is performed by the scientific committee, which is coordinated by the principal investigator. It comprises the researchers associated with the project (co-authors of this paper).

All the departments in the two SCs were assumed to be included, given the agreement obtained by SC senior management on the strength of its hierarchical responsibility. The SC senior chief medical officer is in charge of information to heads of department, and the SC chief nursing officer is responsible for information to front-line managers.

### Data collection and randomisation

The data collection is conducted by two clinical research nurses, two occupational physicists and two psychologist trainees. They are managed by a clinical study coordinator. Staff members are specially recruited for this data collection. Their sole supervisor is the study’s principal investigator.

The quantitative indicators are collected at the time of the mandatory check-up in the occupational medicine department in compliance with French law. This check-up is yearly and thus fits the planned data collections in the study.

After information to front-line managers, all the professionals working in the departments in the two SCs are called on to attend the medical check-up. This call takes the form of an internal letter signed by the head of the occupational medicine department, inviting the person to contact the secretariat to make an appointment. In case on non-response by the agent, two telephone reminders can be made by the secretariat.

The consultations are planned over a period of 3 months at most. The professionals are first seen by a clinical research nurse who presents the research protocol and proceeds to inclusion in the study on the following criteria:

- any professional working in healthcare departments, whatever their profession (chief medical officer, doctor, front-line manager, nurse, nursing assistant, technical staff, secretariat etc)
- written agreement by the professional to take part in the quantitative research.

In case of refusal to participate, the professional is taken to a waiting room until he/she sees the occupational physician for the regulatory, non-standardised check-up.

Professionals agreeing to participate are asked to wait around 40 min in the waiting room and during this time to complete a questionnaire on a tablet. The questionnaire enables the collection of psychometric indicators. The participant is then called by the doctor for a check-up lasting around 35 min. The doctor follows a standardised layout and codes the clinical indicators retrieved from the interview and the clinical examination on a computer. At the end of the consultation the doctor can address the person to an occupational nurse for any complementary biological tests required.

Following the inclusion campaign and baseline data collection, a second random selection is performed by the scientific committee to determine which of the two SCs is the “intervention SC” and which serves as the control.

The collection of indicators at 1 year and 2 years is performed in the same manner.

### The procedure in the intervention group

#### General logic of the intervention

The process we propose concerns the whole system and involves from the outset a transfer of power in the form of a W-QoL negotiation for each unit. Thus, by way of a W-QoL operation coherent with HAS recommendations, all the protagonists from management to healthcare teams directly experiment on an original space for dialogue and co-construction, which supposes 1) for the teams, an increase in scope for action, 2) for the department heads and front-line managers endeavouring to implement a supportive and participative management style and 3) for CHU and SC-level management an arbitration between interests and structural support for teams, without intermediaries.

To facilitate the encounter and to moderate resistance relating to classic roles and relationships, the overall procedure is accompanied for each department by a facilitator (social psychologist or sociologist) following a purpose-designed standardised protocol that was pre-tested in the course of a pilot phase in five CHU units in 2015 and 2016. The facilitators are under the responsibility of the principal investigator and benefit from the supervision of the scientific committee.

We hypothesise that this “experience-generating” procedure on a large scale, agreed to by the senior management and secured and mediated by the scientific protocol is liable to change representations and practices among all the protagonists, for greater empowerment of the medical care teams.

#### Main stages in the intervention

- Step 0. Preparation of the intervention: In the 12 months following confirmation of funding: presentation and validation of the procedure with 1) the CHU General Director, and other directors from the CHU-level management, and 2) the senior management of SCs complying with the inclusion criteria, and finally 3) with the Unions. Creation of the steering committee composed of the directors from the CHU-level management and including the principal investigator and the head of the occupational health department. Invitation to the steering committee to visit an innovating business enterprise recognised in France for its system of piloting based on collective field intelligence. This phase ends with the randomisation and the collection of baseline indicators from the occupational medicine departments. Aim: Top-management acculturation to empowerment, implication of the senior management from the CHU and all the SCs liable to be selected in the random draw, considered as experimentally controlled factors and critical prerequisites for the success of implementation [18].
- Step 1. Start of the intervention: integration of the senior management from the intervention SC into the steering committee and Training seminar n° 1. The seminar intended for department and unit-level management and the senior management of the “intervention SC”, also involving the CHU-level management. This takes place over a half-day, introduced by the DG, the SC senior chief medical officer and SC chief nursing officer, followed by the presentation of the protocol and training on pro-empowerment management. The training includes 1) theoretical contributions on empowerment and management practices, and 2) the intervention of the Chief Executive Officer (CEO) of an enterprise recognised in France for its pro-empowerment management and its level of performance. This CEO shares his personal experience in the transition to empowerment, and then mediates a workshop debate on these practices with the investigator. All the participants are then invited to pursue the exchanges in a friendly manner on the occasion of a buffet. Aim: acculturation and mobilisation of the whole hierarchy for the intervention, promoting the adoption of empowerment practices in the experimental arm.
- Step 2. Qualitative diagnostic phase implicating the various departments and lasting several months. The facilitators meet the department and unit-level managers. They present the system of accompaniment of the healthcare teams. They then carry out exploratory interviews with professionals over a period of two to 3 months. This is followed by the drafting of a qualitative report for each department, covering the resources and constraints perceived by the teams. This written report should not contain any recommendations, so that any proposal for improvement derives from the teams in an empowerment logic. The reports are then validated by the scientific committee. Aims: to facilitate an alliance between facilitators and the front-line management, the department head and the team, and to foster their implication.
- Step 3. Feed-back phase on the reports to the hierarchy, lasting 1 month. The facilitators and the scientific committee hand in all the reports to the steering committee for validation. This is followed by feedback to the department and unit management. Aims: to offer management advice and prepare for the accompaniment of teams with managers at all levels.
- Step 4. Training seminar n°2 intended for management at department level and the management in the “intervention SC”, also involving CHU-level management. As for the first seminar 5 months previously, the event is introduced by the DG, the senior head doctor in the SC and the chief nursing officer. The investigator presents a detailed account of the team accompaniment measures as provided for in the protocol, and the recommended managerial practices. There are new outside contributors, one CEO and one of his staff, belonging to an innovating enterprise recognised in France for its pro-empowerment management and performances. The two contributors present their respective experiences of the same participatory process, and then together mediate a workshop and debate alongside the investigator on the accompaniment and management practices to be instated. All the participants are then invited to pursue the exchanges in friendly manner around a buffet. Aim: to mobilise the whole hierarchical chain towards pro-empowerment management for the implementation of step 5.
- Step 5. Feedback, collective debate on the reports and presentation of the accompaniment plan in each unit, totalling 20 to 23 feedback contributions for the “intervention SC”, over a period of 1 month. The meeting gathers all the healthcare team, the unit-level manager, the head of department, the SC-level managers, the CHU human resource director. After a round-table and presentation of the plan, the facilitator reads out each section of the qualitative analysis, and submits each to validation by the team via collective debate. He/she mediates the debate among protagonists, fostering the emergence of solutions. Finally, he/she proposes a collective accompaniment plan in local working groups in collaboration with management. Aims: to convince of the reality of the negotiation process and implicate the teams in the system aiming to promote empowerment.
- Step 6. Finalisation, launching and accompaniment of the W-QoL working groups in each department, over a period of 3 months. In a post-feedback meeting, the groups are formed by the healthcare teams freely with respect to their composition (number, professions) and their objectives. Working groups or research groups already in existence are integrated into the process. The groups comprise two reference persons – one medical, one paramedical (nurse or nursing assistant), with the back-up of the unit and department management. The head of the department and the unit manager support the reference persons and the groups, and oversee the mutualisation of developments across the different groups, and the overall synthesis in a department perspective. The facilitator has the job of mobilising and supporting the reference persons and the groups, while at the same time providing personalised accompaniment and managerial advice for the head of the department and the unit manager. Once a month, the SC chief nursing officer holds a meeting jointly with the investigator and the front-line managers of the intervention SC on the theme of managerial practices. Aim: in concomitant manner, to foster 1) implication and structural and psychological empowerment in the teams, and 2) the adoption of pro-empowerment managerial practices by department and unit-level managerial staff and SC management.
- Step 7. Negotiation and co-construction meetings in each unit, over a period of 1 month. As for the feedback phase, the meeting assembles the healthcare team, the head nurse, the department head, the SC manager, the human resource manager. The themes broached in this discussion are defined by the working groups in a bottom-up logic. They are made available upstream of the encounter by the investigator to the CHU and SC-level management in the course of a preparatory steering committee meeting. The meetings: Following a round-table, the working group reference persons present the improvements established by the working groups and express any demands for support requiring structural provisions. The CHU and SC-level managerial staff valorise implication and innovations, take an active part in collective reflection on improvements, arbitrate on the scope available to the management, while at the same time informing on the challenges to be dealt with at the overall facility level. The head of the department and the unit manager are encouraged to outline the terms of the meeting and to mediate the exchanges, with the help of the facilitator. Aims: concomitantly to favour 1) implication and psychological and structural empowerment in the teams, 2) the transfer of facilitating functions to unit management and department heads, these being considered as one aspect of pro-empowerment management, and 3) at CHU and SC level, managerial practices that take account of the collective intelligence of the field.
- Step 8. Structural adjustment phase, lasting 2 months. Following the meetings, the managerial bodies implement the decisions and proposals they have made, taking care to comply with undertakings and/or to communicate with the teams in case of unexpected developments. The actions of the management bodies are coordinated in the course of a steering committee meeting gathering the SC managers, the CHU human resource director, the investigator and the facilitators. The SC-level managers are encouraged to return in person to the teams. The facilitators prepare for the cessation of their activities and encourage the department and unit managers to maintain the working groups and their reference persons. Department and unit managers organise team events to take leave of the facilitators, review the accompaniment provided and discuss perspectives for the future. Aims: the start of an improvement loop combining 1) pro-empowerment management in the departments, 2) CHU and SC managerial piloting of performance via negotiation and discussion, and 3) structural and psychological empowerment and team implication.

### The control SC

The “control SC” is merely subject to the annual occupational medicine follow-up, providing the measures at T0, T1 and T2. The participants in the quantitative study carried out at the time of the check-ups are in no way informed of the randomisation to come or the future accompaniment of one of the two SCs. This system makes it possible to dissociate the mandatory health follow-up by occupational medicine from the quantitative study that is common to the two SCs on the one hand, and the collective intervention specific to the “intervention SC” on the other. Thus all participants undertake to be followed up in the long term independently from any intervention.

During the intervention period in the “intervention SC”, the “control SC” functions in the normal manner for the CHU, according to the usual decision-making provisions.

### Criteria for discontinuing intervention

At the individual level, the professionals are free to participate in the intervention. They can therefore decide for themselves to remain outside of the accompaniment. At the unit and department level, the intervention can be interrupted if the head of the department objects to the accompaniment. These complaints are handled by the scientific committee in collaboration with the steering committee.

It should be noted that the two committees each have the power to interrupt intervention in a unit.

### Outcomes

The intervention aims to improve both the structural empowerment and the psychological empowerment of individuals. However, to determine the efficacy of the intervention, and also to understand any effects as accurately as possible, it appears worthwhile assessing conceptual network of empowerment in more global manner [44], here envisaged as predictors and outcomes of empowerment. These are listed in Table 1.

**Table 1:** Key outcomes of the CHRYSALIDE study

Domain	Outcomes *(subdimensions)*	Instruments
Empowerment	Structural empowerment *(opportunity, support, information, resources)*	CWEQII, self-reported questionnaire [45]
	Psychological empowerment *(meaning, autonomy, skills, impact)*	PES, self-reported questionnaire [21]
Motivational processes	Basic psychological needs *(autonomy, competence, social affiliation)*	W-BNS, self-reported questionnaire [46, 47]
	Motivation *(intrinsic, identified, introjected, external motivations, lack of motivation)*	MWMS, self-reported questionnaire [48]
	Affective commitment	ACNC, self-reported questionnaire [49]
Working conditions	Empowering leadership *(delegation of authority, accountability, coaching for self-direction, information-sharing, skill development, coaching for innovation)*	LEBQ, self-reported questionnaire [50]
	Authentic leadership *(transparency, ethics, balanced processing, awareness)*	ALQ, self-reported questionnaire [51]
	Social support *(coworkers, supervisor, and institutional support)*	SPOS, self-reported questionnaire [52, 53]
	Distributive justice *(workload, salary, benefits, responsibilities)*	Organizational justice self-reported scale [54, 55]
	Global justice	Overall justice scale [56, 57]
	Work enrichment and design *(autonomy, feedback, significance, identity, variety of tasks)*	WDQF, self-reported questionnaire [58, 59]
	Role stressors *(role ambiguity, role conflicts)*	RCAS, self-reported questionnaire [60, 61]
	Illegitimate tasks *(unreasonable tasks, unnecessary tasks)*	BITS, self-reported questionnaire [62, 63]
	Quantitative workload	QWI, self-reported questionnaire [64]
	Satisfaction with work schedule	Adapted from usual satisfaction Likert scales [65, 66]
	Legal compliance of the work schedule	Institutional indicator collected by CHU
	Physical exertion at work	Borg 15 points Scale, assessment by practicioner [67]
	Bio-mechanical constraints *(gestures and postures, patient handling activities, activities prior to or following direct patient handling)*	Questionnaire created by the French group of Musculoskeletal Disorders, assessment by practicioner [67, 68]
Health status	Job satisfaction	Likert scale, Self-reported [65]
	Well-being *(pleasure, displeasure, arousal)*	JAWS, Self-reported questionnaire [69]
	Stress	PSS-4 [70, 71]
	Emotional exhaustion	MBI-GS [72]
	Depression	SDI, Diagnosis by practitioner [73]
	Body measurements *(Waist-to-hip ratio, BMI)*	standardized medical assessment
	Heart function *(blood pressure, heart rate)*	standardized medical assessment
	Medical history *(diabetes, high blood pressure, coronary artery disease)*	standardized medical assessment
	Dependent relative at home	standardized medical assessment
	Present medications *(psychotropic, analgesic, diabetes, cholesterol-lowering, antihypertensive treatments)*	standardized medical assessment
	Tobacco consumption	standardized medical assessment
	Alcohol consumption	standardized medical assessment
	Sleep disorders	standardized medical assessment
	Musculoskeletal disorders *(neck, shoulder, elbow, wrist, lumbar)*	standardized medical assessment
	Somatoform disorders	standardized medical assessment
Performance	Absenteeism	Institutional indicator collected by CHU
	Quality of care in the unit	Single-item indicator, self-reported [74]
	Safety of care in the unit	HSOPSc, self-reported questionnaire [75]
	Personal performance at work	HPQ, self-reported questionnaire [76]
	Unit performance	Institutional indicator collected by CHU
	Unit adverse events	Institutional indicator collected by CHU

Psychological empowerment covers cognitions that reflect complex and dynamic motivational processes at work among individuals [12, 15], in terms of 1) internalisation or externalisation of the locus of causality of behaviours in the workplace and 2) actual professional commitment [77]. These processes are also assessed.

Beyond concepts to be measured, the type of measure can entail advantages and drawbacks, for instance depending on whether a questionnaire is self-administered or hetero-administered by a doctor. Likewise, recourse to a standardised medical questionnaire can enable a more reliable evaluation of certain aspects of working conditions, such as biomechanical constraints, and it can diagnose certain health conditions.

### Sample size

Our estimate is based on the effect observed by Dahinten (2014) on the overall score for structural empowerment. Given the hierarchical structure of the data in the present study, (“professional” level nested in the ‘“functional unit” level, in turn nested in the “department” level) an inflation factor needs to be applied to the standard calculation of sample size [78]. The calculation of the inflation factor is based on the following hypotheses:

- an intra-class Correlation Coefficient (ICC) equal to 0.001 (within-SC correlation) We did not find any estimation in the literature of an ICC corresponding to our main judgement criterion and our study population. However ICC values are classically around 0.001 to 0.1 [79]
- an alpha risk of 5%
- a statistical power of 80%
- a difference of 0.12 points between global structural empowerment scores for professionals in the intervention group between T1 and T2 [42]
- a standard deviation of the global structural empowerment score of 0.56 points [42]

With these hypotheses, the number of professionals to include per group is 519. In all, 1038 professionals should be included. This expected number is close to the overall numbers of professionals in the two SCs. Given the considerable risk of loss to follow-up, the choice was to aim for an exhaustive coverage of the two SC, i.e. 600 to 700 individuals per group. We also rely on the compulsory nature of the check-up to reach this objective.

### Statistical analysis

At the outset, the psychometric quality of the concepts measured in the questionnaires will be tested by exploratory and confirmatory factor analysis as well as an exploratory structural equation modelling. These analyses will enable the confirmation of the internal validity of the concepts postulated in the measurement scales and will, if needed, enable the adjustment or rejection of invalid in our sample. The analyses will be conducted under Mplus8 for Windows.

At the individual level, mixed linear models will used to assess the effects of the intervention on each outcome, while taking into account the hierarchical nature of the data, and possible variations in the implementation of the intervention protocol from one department or unit to another. The score of an outcome at T2 is thus explained by the fixed SC effect (intervention versus control) and by the random effect of the department or unit. A statistical adjustment will be made on the outcome score at T0 and on age, gender, profession, working time and size of the team, in line with the literature [36, 80]. The ICC will be estimated to have a precise idea of the effect of the department or unit.

Given the results obtained by Dahinten (2014), which showed a moderating effect of the level of implication at T0 on the impact of the intervention on implication at T1, a second model will be run each time including an interaction terms between the SC group and baseline measures of the outcome variable. These analyses should enable the identification of conditions that favour or undermine the interventions’ effect and implementation.

For the psychometric scales more particularly, the analyses will be performed on both the overall scores and the sub-dimension scores so as to refine the analysis of the effects as far as possible.

At the department and unit level, the score for each outcome will be calculated at T0 and T2 in each group. Mixed-effects linear models will be used to test the effects of the intervention over time, and between departments or units. These analyses will enable the study of variations in the effects of the intervention across the “intervention SC” departments or units.

The alpha risk retained for the statistical analysis is 5%. If the proportion of missing data exceeds 30%, multiple imputation will be considered.

### Data access

The data is collected using an online file completed by the participant or the research staff on computer or tablet. The website and the database are run on the secure Data Management in the CHU. The data collected in the course of the study will be kept in a computerised file in compliance with present French law (*Informatique et libertés,* January 6th 1978, modified in 2004) [81].

The health data of the participants is transferred only to the body to which the researcher in charge of the study is attached, or to any person authorised by him in compliance with confidentiality requirements. The body to which the investigator is attached can request direct access to the medical files to check on procedures and/or the research data, without breaching confidentiality and within the limits of the law. Within the present study, an inspection or audit can be performed. The promoter should be able to grant access to the data for inspectors or auditors. The investigator is to keep all the information relating to the study for at least 15 years after the end of the study.

## Discussion

Empowerment is a key factor of occupational health and organizational performance in care facilities. Nevertheless empowering workers remains a managerial challenge as well as a scientific one.

Empowering workforces implies to improve both structural and psychological empowerment. This means, on the one hand, that environment must provide workers resources, support, opportunity and information and also, on the other hand, that the workers experience autonomy, competence, impact and meaning. The purpose is to internalize motivation and finally improve commitment, health and performance.

But these processes need a real transformation of managerial practices from the top to first-line management level, for the purpose of sustainably and truly enhancing the powers of workers [18, 82].

Therefore, the main strength of the CHRYSALIDE study consists of the intervention design which combines accompaniment of healthcare teams with training and accompaniment of front-line managers and training and accompaniment of SC-level managers [83].

The results of this study will contribute to address the need for concrete methodology to empower workforces in care facilities, by transforming managerial practices of all the hierarchical strata [82, 84].

With this aim, the series of measurements assesses a wide range of the empowerment predictors and outcomes and also mediators (such as motivational processes), while combining self and hetero-evaluation, enabling strong measures and precise analysis of the effects of the intervention and enriching discussions for the design of future interventional studies [44].

Furthermore, measurements are made during mandatory medical check-ups in the Occupational Medicine Department of the CHU. This recruitment strategy should enhance follow-up at 2 years.

Obviously the implementation and the results of the analysis of the effects of the intervention at one and 2 years will be reported in a series of scientific articles. In addition to the scientific valorisation, the results will also be circulated to the public authorities and the managerial community of health facilities, at least in France. We hope the results will contribute to reflection on prevention and management policies, and to the development of W-QoL. If the intervention is a success, the system will warrant replication in other SCs and in other health facilities.

## Data Availability

The dataset collected in the study will be available from the corresponding author on reasonable request after 3 years from the end of the study.

## Abbreviations

ACNC: Affective, Continuance and Normative Commitment
ALQ: Authentic leadership Questionnaire
BITS: Bern Illegitimate Tasks Scale
CEO: Chief Executive Officer
CHU: University Hospital Complex
CWEQII: Conditions for Work Effectiveness Questionnaire two
DG: General Director
HAS: French health authority
HPQ: World Health Organization Health and Work Performance Questionnaire
HSOPSc: Hospital Survey On Patient Safety culture
ICC: Intra-class Correlation Coefficient
JAWS: Job Affective Well-being Scale
LEBQ: Leader Empowering Behaviours Scale
MBI-GS: Maslach Burnout Inventory-General Survey
MWMS: Multidimensional Work Motivation Scale
PES: Psychological Empowerment Scale
PSS: Perceived Stress Scale
QWI: Quantitative workload inventory
RCAS: Role Conflicts and Ambiguity Scale
SC: Sub-Center
SDI: Short Depression Interview
SPOS: Survey of Perceived Organizational Support
W-BNS: Work Basic Need Satisfaction
WDQF: Work Design Questionnaire in French
W-QoL: Workplace Quality-of-Life
